# Acute Variceal Bleeding: Somatostatin or Sclerotherapy?

**DOI:** 10.1155/1994/41079

**Published:** 1994

**Authors:** Jacob Korula

**Affiliations:** Liver Unit University of Southern California Rancho Los Amigos Medical Center Downey California USA


enucleation was employed had an operative blood loss exceeding 1400 mL. Depending upon intraoperative hemodynamics, blood products were pro-bably transfused in some patients. Albeit infrequent, transfusion related risks can be life threatening. Ideally a surgical technique for benign disease should eliminate any transfusion risk when employed. Did the adjuncts used herein reduce blood loss? Unfortunately the data presented by Baer *et al.*, leaves this question unanswered because there are no data for comparison. Is the rationale for arterial ligation sound? Substantiation that preoperative angiography identified the exact arterial supply to the hemangioma would support their contention that extrahepatic ligation of the major hepatic arterial branch to the hemangioma is advantageous. However, that data was not presented. Moreover the concept is suspect because the classic arterial supply to hepatic hemangiomas is through multiple small peripheral arteries rather than a single dominant branch arising extrahepatically from a main lobar hepatic artery. If heman-giomas are enucleated, the arterial supply will be ligated immediately adjacent to the hemangioma anyway. Whether extrahepatic ligation confers any additional benefit is unknown. Finally Baer *et al.*, favor intermittent vascular inflow occlusion to the liver during enucleation. Unless the time for enucleation of the hemangioma from the parenchyma routinely exceeded 45–60 minutes, continuous rather than intermittent occlusion would likely be more effective in reducing blood loss because intermittent perfusion of the interface would be avoided.

Although the exact technique for resection of hemangiomas may not need to be so elaborate, the concept of broader application of local resection or enucleation is laudable. Utilization of the plane of compressed liver parenchyma adjacent to the hemangioma during continuous inflow vascular occlusion should allow enucleation to be performed safely without sacrifice of adjacent normal liver and without any permanent liver ischemia. The technique of Baer *et al.*, should be heeded. Refinements will follow.

David M. Nagorney
Mayo Clinic
200 First Street SW, Rochester
MN 55905 U.S.A

### ACUTE VARICEAL BLEEDING: SOMATOSTATIN OR SCLEROTHERAPY?

### ABSTRACT

*Shields, R., Jenkins, S. A., Baxter, J. N., Kingsnorth, A. N., Ellenbogen, S. A., Makin, C.A., Gilmore, I., Morris, A. I., Ashby, D. and West, C. R. (1992) A prospective randomised controlled trial comparing the efficacy of somatostatin with injection sclerotherapy in the control of bleeding oesophageal varices. Journal of Hepatology 16, 128–137.*

**Since previous reports have suggested that somatostatin may be of value in the control of acute variceal haemorrhage, we compared its efficacy with that of injection sclerotherapy in a randomised controlled clinical trial. Eighty consecutive patients with endoscopically-proven severe variceal bleeding were randomised to injection sclerotherapy ($n = 41$) or somatostatin ($n = 39$) given as a continuous infusion of 250 μg/h for 5 days plus daily bolus administration of 250 μg. The efficacy of injection sclerotherapy and somatostatin infusion in controlling haemorrhage and preventing rebleeding (censored at 5 days), mortality (censored at 28 days) and complications was compared. The aetiology of the portal hypertension and transfusion requirements was similar between the two groups, but there were more patients with severe liver disease (Child's C) in the somatostatin group.**

**There was no significant difference between the two treatments in the initial ($p = 1.0$) or overall control of bleeding ($p = 0.58$). Furthermore, somatostatin was as effective as injection sclerotherapy in cotrolling bleeding in patients with severe liver disease or in those actively bleeding at the time of their endoscopy. The relative risk of rebleeding whilst receiving somatostatin compared to injection sclerotherapy was 1.39 [95% Confidence Interval (CI) 3.73; 0.52], but this was reduced to 0.98 (95% CI 0.37; 2.67) when readjusted for Child's grading, the only prognostic factor shown to be of significance. Mortality was not significantly different between the two groups of patients ($p = 0.31$). The relative risk of dying whilst receiving somatostatin compared to injection sclerotherapy was 1.6 (95% CI 3.93; 0.66) but was reduced to 1.03 (95% CI 0.47; 2.47) when adjusted for Child's grading, the only significant prognostic factor. Complications in the somatostatin group were minor and less frequent than after injection sclerotherapy. The results of this study indicate that somatostatin is a safe treatment, which is as effective as endoscopic injection sclerotherapy for acute variceal bleeding.**

KEY WORDS: Portal hypertension, oesophageal varices, injection sclerotherapy, somatostatin, acute variceal bleeding

### PAPER DISCUSSION

This paper is another in a series of clinical studies attempting to determine the efficacy of intravenous somatostatin for the treatment of bleeding esophageal varices. Somatostatin is a 14 aminoacid peptide that reduces splanchnic blood flow[1] leading to a modest reduction in hepatic blood flow and wedged hepatic venous pressure,[2] with little effect on portal pressure[3] and intravariceal pressure[4]. Interest in this drug for therapy of variceal hemorrhage developed because of few side effects and comparable efficacy to vasopressin. The costs of treatment however are significantly greater.

This study is different because comparison is made with endoscopic sclerotherapy, recognized to be effective treatment for control of acute variceal hemorrhage[5]. In evaluating this study in the context of previously published reports, we need to examine the design of the study. Patients with hemodynamically significant variceal bleed were enrolled following an endoscopy performed as "soon as possible" after admission. Of the 111 patients referred for treatment, 80 patients fulfilled their criteria and were randomized to either therapy. Somatostatin was infused uninterrupted for 5 days at 250 ug/h after a bolus of 250 ug; the bolus was repeated each day and when infusion was thought to be interrupted while changing infusion bags. Balloon tamponade was offered to patients if bleeding was severe and was kept in place for 12 hours or until definitive therapy was planned. Patients randomized to endoscopic sclerotherapy received intravariceal injections of 2–3 ml of 5% ethanolamine oleate into each variceal column and with the help of an overtube advanced over a fiberoptic endoscope. Balloon tamponade was placed for initial control of bleeding and if oozing persisted after sclerotherapy. In both groups of patients, the re-insertion of the balloon and continued bleeding with or without hemodynamic instability constituted treatment failure. At the end of 5 days, definitive therapy was commenced and efficacy of the two treatments determined by assessment of 28 day mortality and treatment related complications. Although the frequency is not stated, the use of balloon tamponade in this study to initially stabilize patients and for rebleeding while on somatostatin infusion or after sclerotherapy confirms the reliance of this group on the beneficial effects of tamponade. Therefore, in evaluating the efficacy of the two treatments under study, we must interpret the results as showing the combined effect of balloon tamponade with either of these modalities.

The patient groups were well matched in terms of age, gender, aetiology of liver disease, severity of liver disease, index versus interval bleed, blood transfusion requirement before and during the treatment and the time that elapsed between referral, randomization and inception of therapy. What is truly remarkable is that the average time from onset of the bleed to hospital admission was only 1.5 to 2 hours, and the average time from onset of bleed to start of treatment was 4 to 5 hours. These time related variables would be difficult to reproduce in most centers and certainly attests to the vigilance of this research team to respond to a variceal bleeder with alarcity.

The results of this study however are interesting. Endoscopic sclerotherapy controlled bleeding in 98%. Six of the 40 patients (15%) who rebled within 5 days were treated with balloon tamponade and somatostatin; overall bleeding control was 83%. In the somatostatin group, initial control of hemorrhage was 97%, overall bleeding control was 77% and 8 patients (21%) rebled. Seven of these received balloon tamponade, 3 of whom also received sclerotherapy and all died following emergency esophageal transection. Although rebleeding rates were similar in the two groups, the choice of alternative treatment for either group was different. For instance, patients in the sclerotherapy group were offered balloon tamponade and somatostatin rather than additional sclerotherapy treatments or surgery. In the somatostatin group, all patients received balloon tamponade, some received sclero- therapy and eventually surgery was performed in all. It is difficult therefore to assess the effect of the two treatments on outcome when decisions regarding alternative and definitive treatment were not consistent.

While their results of endoscopic sclerotherapy in initial control of hemorrhage, rebleeding and mortality are similar to published reports,[6] it is not clear why sclerotherapy failed in the somatostatin patients who rebled. Sclerotherapy was effective in only 1 of these 7 patients (15%), a significantly lower rate than their initial sclerotherapy control rate of 98%. Why should prior infusion of somatostatin contri-bute to decreased efficacy of sclerotherapy? Did these patients have more severe liver disease or become decompensated with uncontrolled bleeding? Such a possibility is plausible since all 7 patients who received emergency esophageal transection died. On the other hand the initial control of bleeding with somatostatin is somewhat higher than seen in the large clinical controlled studies where overall success of 64 to 68% were reported[7–9]. In these studies the efficacy of somatostatin was not consistently beneficial since cessation of bleeding in controls occurred as high as 83%[8]. What is puzzling is why any effect on control of bleeding can be expected when this drug has minimal effects on portal pressure and intravariceal pressure.

The authors of this study conclude that somatostatin should be offered to all patients as the initial therapy for variceal bleeding since it's efficacy equals that of endoscopic sclerotherapy and because of it's easy administration. While there is merit in this recommendation, one should question whether somatostatin need be offered as an alternative to definitive therapy. Should patients be continued on this drug for 5 days as suggested in this study and by Burroughs *et al.*?[9] Or should somatostatin be used primarily to stabilize patients initially while definitive therapy is being arranged or organized? Ultimately, treatment decisions will be based on the time of patient presentation, condition of the patient and the radiologic, surgical and endoscopic expertise available at each center. For a start anyway and for the initial management of variceal hemorrhage, somatostatin seems good treatment.

### *References*

1. Sonnenberg, G. E., Keller, U. and Perruchud, A. *et al.* (1981) Effect of somatostatin on splachnic hemo-dynamics in patients with cirrhosis of the liver and in normal subjects. *Gastroenterology*, **80**, 526–532
2. Bosch, J., Kravetz, D. and Rodes, J. (1981) Effects of somatostatin on hepatic and systemic hemodynamics in patients with cirrhosis of the liver. Comparison with vasopressin. *Gastroenterology*, **80**, 518–525
3. Sonnenberg, A. and West, C. (1983) Somatostatin reduces gastric mucosal blood flow in normal subjects but not in patients with cirrhosis of the liver. *Gut*, **24**, 148–153
4. Kleber, G., Sauerbruch, T., Fischer, G. and Paumgartner, G. (1988) Somatostatin does not reduce oesophgaeal variceal pressure in liver cirrhotics. *Gut*, **29**, 153–156
5. Larson, A. W., Cohen, H. and Zweiban, B. *et al.* (1989) Acute esophageal variceal sclerotherapy. Results of a prospective randomized controlled trial. *JAMA*, **255**, 497–500
6. Terblanche, J., Burroughs, A. K. and Hobbs, K. E. F. (1989) Controversies in the management of bleeding esophageal varices. *N. Eng. J. Med.*, **320**, 1469–1475
7. Loperfido, S., Godena, F. and Tosolini, G. *et al.* (1987) La somatatostatina nel tratamento dell'emorraga da varici esofagogastriche. *Recenti Prog. Med.*, **78**, 82–86
8. Valenzuela, J. E., Schubert, T. and Fogel, M. R. *et al.* (1989) A multicenter randomized double-blind trial of somatostatin in the management of acute hemorrhage from esophageal varices. *Hepatology*, **10**, 958–961
9. Burroughs, A. K., McCormick, P. A. and Hughes, M. D. *et al.* (1990) Randomized double-blind placebo controlled study of somatostatin for control of variceal bleeding. *Gastroenterology*, **99**, 1388–1395

Jacob Korula
Liver Unit
University of Southern California
Rancho Los Amigos Medical Center
Downey, California, USA

